# Crystal structure and Hirshfeld surface analysis of (4*Z*)-1-butyl-4-(2-oxo­propyl­idene)-2,3,4,5-tetra­hydro-1*H*-1,5-benzodiazepin-2-one

**DOI:** 10.1107/S2056989018014779

**Published:** 2018-10-26

**Authors:** Karim Chkirate, Nada Kheira Sebbar, Tuncer Hökelek, Damodaran Krishnan, Joel T. Mague, El Mokhtar Essassi

**Affiliations:** a Laboratoire de Chimie Organique Hétérocyclique URAC 21, Pôle de Compétence Pharmacochimie, Av. Ibn Battouta, BP 1014, Faculté des Sciences, Université Mohammed V, Rabat, Morocco; b Laboratoire de Chimie Bioorganique Appliquée, Faculté des sciences, Université Ibn Zohr, Agadir, Morocco; c Department of Physics, Hacettepe University, 06800 Beytepe, Ankara, Turkey; d Department of Chemistry, University of Pittsburgh, Pennsylvania, PA 15260, USA; e Department of Chemistry, Tulane University, New Orleans, LA 70118, USA

**Keywords:** crystal structure, benzodiazepine, hydrogen bond, π-stacking, Hirshfeld surface

## Abstract

The asymmetric unit of the title compound, C_16_H_20_N_2_O_2_, consists of two independent mol­ecules differing slightly in the conformations of the seven-membered rings and the butyl substituents, where the benzene rings are oriented at a dihedral angle of 34.56 (3)°. In the crystal, pairwise inter­molecular C—H⋯O and complementary intra­molecular C—H⋯O hydrogen bonds form twisted strips extending parallel to (012). These strips are connected into layers parallel to (111) by additional inter­molecular C—H⋯O hydrogen bonds. The layers are further joined by C—H⋯π inter­actions. The Hirshfeld surface analysis of the crystal structure indicates that the most important contributions for the crystal packing are from H⋯H (65.5%), H⋯C/C⋯H (16.0%) and H⋯O/O⋯H (15.8%) inter­actions.

## Abstract 2

The asymmetric unit of the title compound consists of two independent mol­ecules differing slightly in the conformations of the seven-membered rings and the butyl substituents.

## Chemical context   

**Figure 1:**
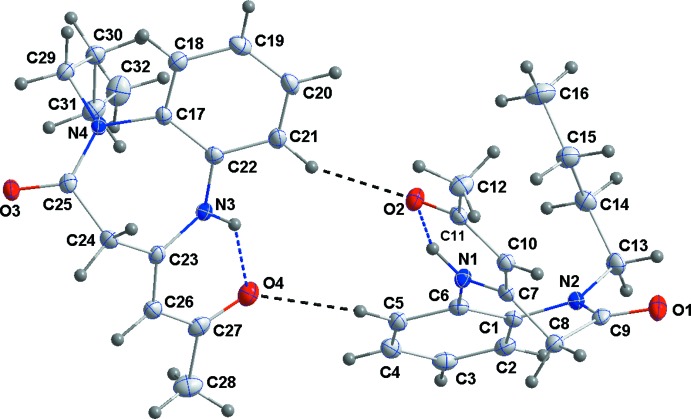
The asymmetric unit with the labelling scheme and 50% probability ellipsoids. N—H⋯O and C—H⋯O hydrogen bonds are indicated by blue and black dashed lines, respectively.

1,5-Benzodiazepine derivatives constituted an important class of heterocyclic compounds possessing a wide spectrum of biological properties. They exhibit anti-inflammatory (Roma *et al.*, 1991 ▸), hypnotic (Kudo *et al.*, 1982 ▸), anti-HIV-1 (Di Braccio *et al.*, 2001 ▸), anti­convulsant (De Sarro *et al.*, 1996 ▸), anti­microbial (Kumar *et al.*, 2007 ▸) and anti­tumor (Kamal *et al.*, 2008 ▸) activities. The present work is a continuation of the synthesis of the N-substituted 1,5-benzodiazepines derivatives performed recently by our team (Sebhaoui *et al.*, 2016 ▸, 2017 ▸; Chkirate *et al.*, 2018 ▸). In this work, we prepared the title compound, for an investigation of its biological activities, by reacting (*Z*)-4-(2-oxo­propyl­idene)-4,5-di­hydro-1*H*-benzo[*b*][1,5]diazepin-2(3*H*)-one with 1-bromo­butane, under liquid–liquid phase-transfer catalysis (PTC) conditions using tetra *n*-butyl ammonium bromide (TBAB) as catalyst and an aqueous solution of potassium hydroxide as base in di­chloro­methane (Fig. 1 ▸). We report herein its crystal and mol­ecular structures along with the Hirshfeld surface analysis.

## Structural commentary   

The asymmetric unit of the title compound consists of two independent mol­ecules differing modestly in the conformations of the seven-membered *B* (N1/N2/C1/C6–C9) and *D* (N3/N4/C17/C22–C25) rings and the *n*-butyl substituents, where the benzene *A* (C1–C6) and *C* (C17–C22) rings are oriented at a dihedral angle of 34.56 (3)°. Rings *B* and *D* have boat conformations with slightly different Cremer–Pople puckering parameters [for ring *B*: *Q*(2) = 0.8872 (13) Å, *Q*(3) = 0.2030 (13) Å, φ(2) = 28.49 (8)° and φ(3) = 138.6 (4)°, *Q*_T_ = 0.9102 (13) Å and for ring *D*: *Q*(2) = 0.8631 (13) Å, *Q*(3) = 0.2113 (13) Å, φ(2) = 24.61 (8)° and φ(3) = 136.8 (3)°, *Q*_T_ = 0.8886 (13) Å]. In the *n*-butyl substituents, the C13—C14—C15—C16 [177.96 (13)°] and C29—C30—C31—C32 [174.97 (12)°] chains also have slightly different torsion angles. The conformation of the 2-oxo­propyl­idene units are partially determined by the intra­molecular N—H⋯O hydrogen bonds (Table 1 ▸, Fig. 1 ▸) The r.m.s. deviation of the overlay of two molecules is 0.1367 Å.
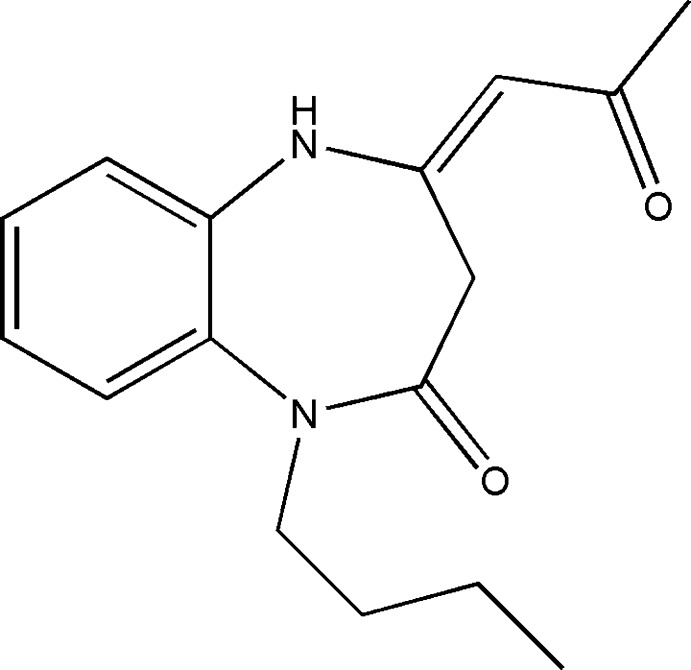


## Supra­molecular features   

**Figure 2:**
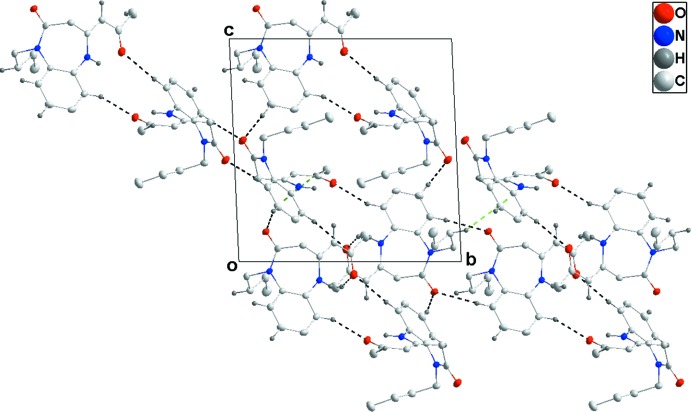
Detail of inter­molecular C—H⋯O hydrogen bonding (black dashed lines) and C—H⋯π (ring) inter­actions (green dashed lines) viewed along the *a*-axis direction.

**Figure 3:**
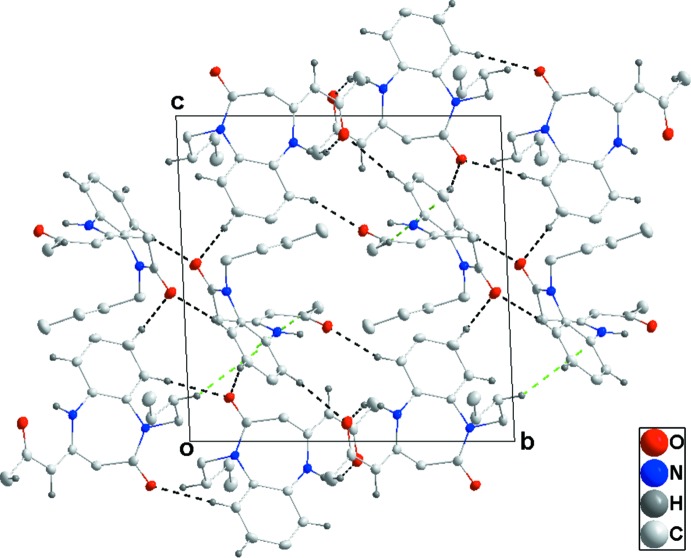
Packing viewed along the *a*-axis direction with inter­molecular inter­actions depicted as in Fig. 2 ▸.

Hydrogen bonding and van der Waals contacts are the dominant inter­actions in the crystal packing. In the crystal, pairwise inter­molecular C—H_Bnz_⋯O_Oxoprp_ (Bnz = Benzene and Oxoprp = 2-oxo­propyl­idene) and complementary intra­molecular C—H_Bnz_⋯O_Bnzdzp_ (Bnzdzp = 1,5-benzodiazepin-2-one) hydrogen bonds (Table 1 ▸) form twisted strips extending parallel to (012) (Fig. 2 ▸). These strips are connected into layers parallel to (111) (Fig. 3 ▸) by inter­molecular C—H_Bnz_⋯O_Oxoprp_ and C—H_Bnzdzp_⋯O_Bnzdzp_ hydrogen bonds (Table 1 ▸). The layers are further joined by C—H_Bnzdzp_⋯π and C—H_Bty_⋯π (Bty = *n*-but­yl) inter­actions (Table 1 ▸ and Figs. 2 ▸ and 3 ▸).

## Hirshfeld surface analysis   

**Figure 4:**
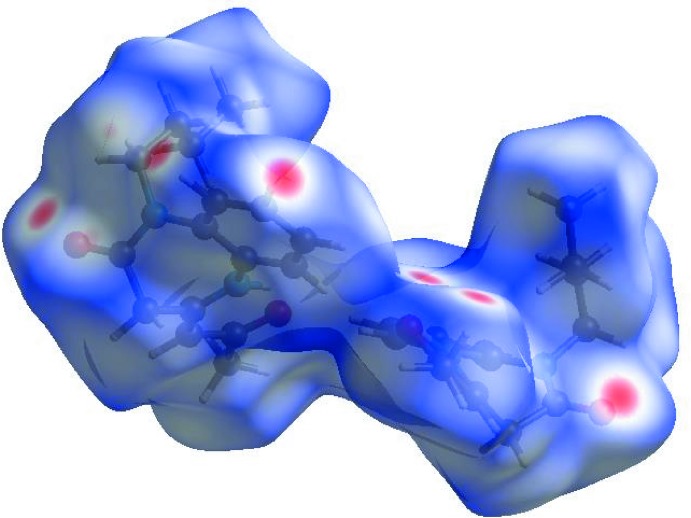
View of the three-dimensional Hirshfeld surface of the title compound plotted over *d*_norm_ in the range −0.2745 to 1.3634 a.u.

**Figure 5:**
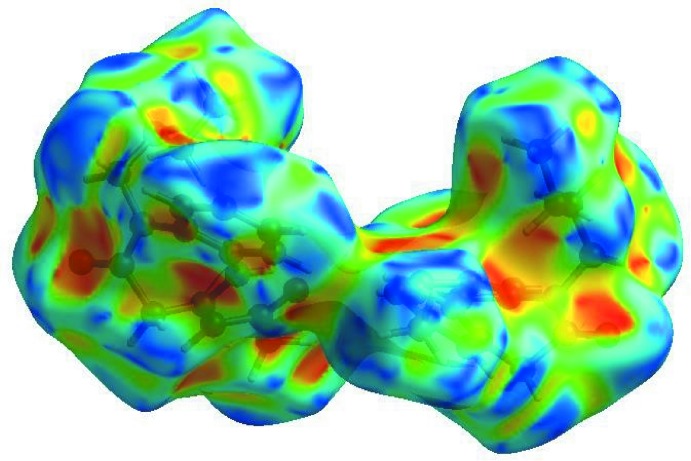
Hirshfeld surface of the title compound plotted over shape-index.

In order to visualize the inter­molecular inter­actions in the crystal of the title compound, a Hirshfeld surface (HS) analysis (Hirshfeld, 1977 ▸; Spackman & Jayatilaka, 2009 ▸) was carried out by using *Crystal Explorer17.5* (Turner *et al.*, 2017 ▸). In the HS plotted over *d*_norm_ (Fig. 4 ▸), the white surface indicates contacts with distances equal to the sum of van der Waals radii, and the red and blue colours indicate distances shorter (in close contact) or longer (distinct contact) than the van der Waals radii, respectively (Venkatesan *et al.*, 2016 ▸). The bright-red spots appearing near O1, O2, O3 and hydrogen atoms H18, H19 and H28*C* indicate their roles as the respective donors and acceptors in the dominant C—H⋯O and N—H⋯O hydrogen bonds. The shape-index of the HS is a tool for visualizing π–π stacking inter­actions by the presence of adjacent red and blue triangles; if there are no adjacent red and/or blue triangles, then there are no π–π inter­actions. Fig. 5 ▸ clearly suggests that there are no π–π inter­actions.

**Figure 6:**
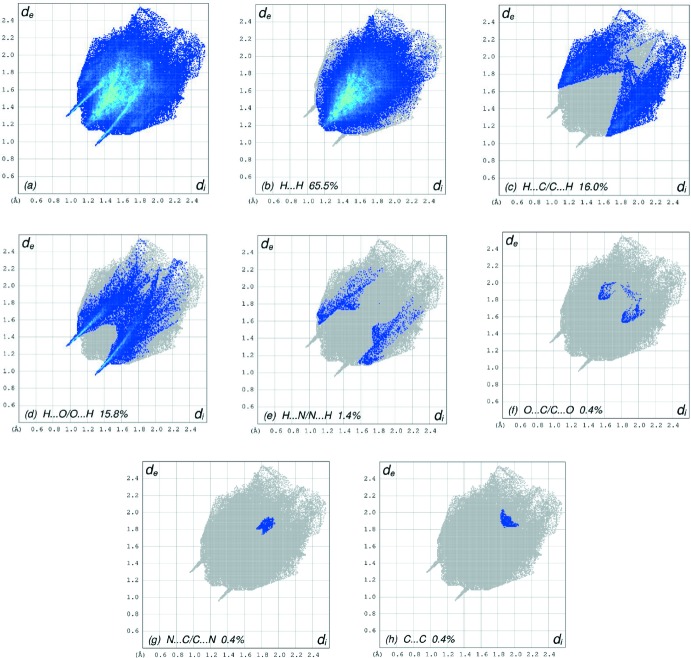
The full two-dimensional fingerprint plots for the title compound, showing (*a*) all inter­actions, and delineated into (*b*) H⋯H, (*c*) H⋯C/C⋯H, (*d*) H⋯O/O⋯H, (*e*) H⋯N/N⋯H, (*f*) O⋯C/C⋯O, (*g*) N⋯C/C⋯N and (*h*) C⋯C inter­actions. *d*_i_ and *d*_e_ are the closest inter­nal and external distances (in Å) from given points on the Hirshfeld surface.

**Figure 7:**
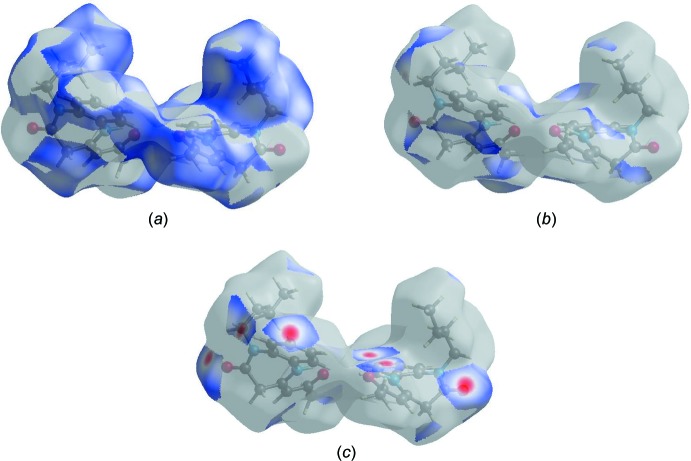
Hirshfeld surface representations of *d*_norm_ for (*a*) H⋯H, (*b*) H⋯C/C⋯H and (*c*) H⋯O/O⋯H inter­actions.

The overall two-dimensional fingerprint plot, Fig. 6 ▸ *a*, and those delineated into H⋯H, H⋯C/C⋯H, H⋯O/O⋯H, H⋯N/N⋯H, O⋯C/C⋯O, N⋯C/C⋯N and C⋯C contacts (McKinnon *et al.*, 2007 ▸) are illustrated in Fig. 6 ▸ *b*–*h*, respectively, together with their relative contributions to the Hirshfeld surface. The most important inter­action is H⋯H contributing 65.5% to the overall crystal packing, which is reflected in Fig. 6 ▸ *b* as widely scattered points of high density due to the large hydrogen-atom content of the mol­ecule. The wide peak in the centre at *d*_e_ = *d*_i_ = 1.16 Å in Fig. 6 ▸ *b* is due to the short inter­atomic H⋯H contacts (Table 2 ▸). In the presence of weak C—H⋯π inter­actions (Table 1 ▸) in the crystal, the pair of characteristic wings resulting in the fingerprint plot delineated into H⋯C/C⋯H contacts, Fig. 6 ▸ *c*, the 16.0% contribution to the HS is viewed as pair of spikes with the tips at *d*_e_ + *d*_i_ ∼ 2.73 Å. The H⋯O/O⋯H contacts in the structure, with 15.8% contribution to the HS, have a symmetrical distribution of points, Fig. 6 ▸ *d*, with the tips at *d*_e_ + *d*_i_ ∼2.24 Å arising from the short intra- and/or inter­atomic C—H⋯O and N—H⋯O hydrogen bonding (Table 1 ▸) as well as from the H⋯O/O⋯H contacts (Table 2 ▸). Finally, the H⋯N/N⋯H (Fig. 6 ▸ *e*) contacts (Table 2 ▸) in the structure, with a 1.4% contribution to the HS, have a symmetrical distribution of points, with a pair of wings appearing at *d*_e_ = *d*_i_ = 2.67 Å. The Hirshfeld surface representations for *d*_norm_ are shown for the H⋯H, H⋯C/C⋯H and H⋯O/O⋯H inter­actions in Fig. 7 ▸ *a*–*c*, respectively.

The Hirshfeld surface analysis confirms the importance of H-atom contacts in establishing the packing. The large number of H⋯H, H⋯C/C⋯H and H⋯O/O⋯H inter­actions suggest that van der Waals inter­actions and hydrogen bonding play the major roles in the crystal packing (Hathwar *et al.*, 2015 ▸).

## Synthesis and crystallization   

To a solution of (*Z*)-4-(2-oxo­propyl­idene)-4,5-di­hydro-1*H*-benzo[*b*][1,5]diazepin-2(3*H*)-one (2.38 mmol) in 15 ml of di­chloro­methane were added 1.5 eq of 1-bromo­butane, (3.57 mmol) of potassium hydroxide dissolved in water and 0.23 mmol of tetra-*n*-butyl ammonium bromide (BTBA). The mixture was kept under magnetic stirring at room temperature for 48 h. A little water was added and then the organic phase was extracted. The mixture obtained was chromatographed on a column of silica gel (eluent hexa­ne/ethyl acetate 8/2) to give three products. The title compound was isolated as the major product in a yield of 77%.

## Refinement   

Crystal data, data collection and structure refinement details are summarized in Table 3 ▸. H atoms attached to C28 did not give a satisfactory geometry so they were positioned geometrically with C—H = 0.98 Å, and refined as riding with *U*_iso_(H) = 1.5*U*_eq_(C). The remaining H atoms were located in a difference-Fourier map and were freely refined. The crystal studied was twinned.

## Supplementary Material

Crystal structure: contains datablock(s) I, global. DOI: 10.1107/S2056989018014779/xu5946sup1.cif

Structure factors: contains datablock(s) I. DOI: 10.1107/S2056989018014779/xu5946Isup2.hkl

Supporting information file. DOI: 10.1107/S2056989018014779/xu5946Isup3.cdx

Supporting information file. DOI: 10.1107/S2056989018014779/xu5946Isup4.cml

CCDC reference: 1874203

Additional supporting information:  crystallographic information; 3D view; checkCIF report

## Figures and Tables

**Table 1:** Hydrogen-bond geometry (Å, °) *Cg*1 is the centroid of benzene ring *A* (C1–C6).

*D*—H⋯*A*	*D*—H	H⋯*A*	*D*⋯*A*	*D*—H⋯*A*
N1—H1⋯O2	0.927 (17)	1.834 (17)	2.5998 (14)	138.2 (13)
N3—H3*A*⋯O4	0.898 (17)	1.901 (17)	2.6349 (14)	137.6 (14)
C2—H2⋯O1^ii^	0.964 (15)	2.469 (16)	3.4235 (17)	170.6 (11)
C3—H3⋯O3^vi^	0.968 (15)	2.420 (17)	3.3714 (16)	166.0 (11)
C5—H5⋯O4	0.998 (16)	2.456 (15)	3.4086 (17)	159.3 (11)
C18—H18⋯O3^v^	0.961 (14)	2.556 (15)	3.5165 (16)	176.4 (11)
C19—H19⋯O1^i^	1.001 (15)	2.330 (15)	3.3273 (15)	177.0 (12)
C21—H21⋯O2	0.986 (15)	2.277 (15)	3.1933 (16)	154.1 (11)
C28—H28*C*⋯O4^vi^	0.98	2.48	3.4342 (18)	164
C12—H12*A*⋯*Cg*1^x^	0.999 (19)	2.921 (19)	3.9047 (16)	167.8 (13)
C30—H30*A*⋯*Cg*1^xii^	1.007 (16)	2.903 (15)	3.8016 (15)	149.0 (11)

Symmetry codes: (i) 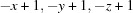; (ii) 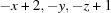; (v) 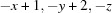; (vi) 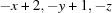; (x) 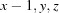; (xii) 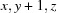.

**Table 2:** Selected interatomic distances (Å)

O1⋯H19^i^	2.328 (16)	C11⋯H26^iv^	2.976 (14)
O1⋯H13*A* ^ii^	2.878 (18)	C13⋯H2	2.746 (15)
O1⋯H2^ii^	2.468 (15)	C17⋯H24*B*	2.635 (14)
O1⋯H13*B*	2.242 (15)	C17⋯H30*B*	2.810 (15)
O1⋯H8*B* ^iii^	2.858 (15)	C18⋯H29*B*	2.688 (14)
O2⋯H21	2.277 (15)	C21⋯H14*B* ^ix^	2.961 (17)
O2⋯H12*C* ^i^	2.627 (18)	C21⋯H28*A* ^iv^	2.94
O2⋯H1	1.834 (17)	C22⋯H24*B*	2.679 (14)
O2⋯H26^iv^	2.780 (14)	C25⋯H29*B* ^v^	2.815 (14)
O3⋯H18^v^	2.556 (15)	C26⋯H4^vi^	2.988 (16)
O3⋯H3^vi^	2.424 (15)	C27⋯H3*A*	2.459 (17)
O3⋯H29*B* ^v^	2.637 (15)	C29⋯H18	2.768 (15)
O3⋯H29*A*	2.300 (14)	H1⋯H5	2.48 (2)
O4⋯H3*A*	1.901 (18)	H2⋯H13*A*	2.26 (2)
O4⋯H24*B* ^iv^	2.761 (14)	H2⋯H13*B* ^ii^	2.51 (2)
O4⋯H5	2.456 (15)	H3*A*⋯H21	2.39 (2)
O4⋯H28*C* ^vi^	2.48	H8*B*⋯H10	2.40 (2)
N1⋯H24*A* ^iv^	2.775 (15)	H10⋯H12*A*	2.50 (2)
N1⋯H15*A*	2.858 (17)	H12*A*⋯H14*A* ^x^	2.49 (2)
N2⋯H13*A* ^ii^	2.828 (16)	H13*B*⋯H32*A* ^ix^	2.54 (2)
N2⋯H15*A*	2.704 (16)	H13*B*⋯H15*B*	2.57 (2)
N3⋯H31*B*	2.915 (17)	H14*A*⋯H16*A*	2.52 (2)
N4⋯H31*B*	2.705 (17)	H15*B*⋯H32*A* ^ix^	2.54 (2)
C1⋯H8*A*	2.669 (16)	H18⋯H29*B*	2.21 (2)
C1⋯H14*A*	2.818 (15)	H24*A*⋯H26	2.34 (2)
C2⋯H13*A*	2.790 (16)	H26⋯H28*A*	2.33
C2⋯H30*A* ^vii^	2.970 (14)	H28*B*⋯H32*C* ^vi^	2.50
C3⋯H12*A* ^viii^	2.833 (19)	H29*A*⋯H31*A*	2.54 (2)
C3⋯H30*A* ^vii^	2.852 (15)	H29*A*⋯H32*B* ^xi^	2.58 (2)
C6⋯H8*A*	2.652 (15)	H30*A*⋯H32*B*	2.50 (2)
C9⋯H13*A* ^ii^	2.828 (17)	H31*A*⋯H31*A* ^xi^	2.55 (2)
C10⋯H20^i^	2.871 (15)	H31*A*⋯H32*B* ^xi^	2.57 (2)
C11⋯H1	2.424 (17)	H32*C*⋯H28*B* ^vi^	2.50

Symmetry codes: (i) 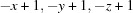; (ii) 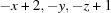; (iii) 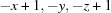; (iv) 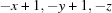; (v) 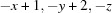; (vi) 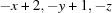; (vii) 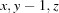; (viii) 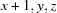; (ix) 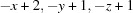; (x) 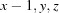; (xi) 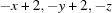.

**Table 3:** Experimental details

Crystal data	
Chemical formula	C_16_H_20_N_2_O_2_
*M*_r_	272.34
Crystal system, space group	Triclinic, *P* 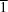
Temperature (K)	100
*a*, *b*, *c* (Å)	9.1132 (6), 12.6676 (9), 12.8164 (9)
α, β, γ (°)	91.344 (1), 99.537 (1), 96.340 (1)
*V* (Å^3^)	1448.87 (17)
*Z*	4
Radiation type	Mo *K*α
μ (mm^−1^)	0.08
Crystal size (mm)	0.34 × 0.29 × 0.25

Data collection	
Diffractometer	Bruker SMART APEX CCD
Absorption correction	Multi-scan (*TWINABS*; Sheldrick, 2009 ▸)
*T*_min_, *T*_max_	0.97, 0.98
No. of measured, independent and observed [*I* > 2σ(*I*)] reflections	51542, 51542, 40020
*R*_int_	0.029
(sin θ/λ)_max_ (Å^−1^)	0.696

Refinement	
*R*[*F* ^2^ > 2σ(*F* ^2^)], *wR*(*F* ^2^), *S*	0.045, 0.133, 1.08
No. of reflections	51542
No. of parameters	511
H-atom treatment	H atoms treated by a mixture of independent and constrained refinement
Δρ_max_, Δρ_min_ (e Å^−3^)	0.41, −0.32

Computer programs: *APEX3* and *SAINT* (Bruker, 2016 ▸), *SHELXT* (Sheldrick, 2015*a* ▸), *SHELXL2018* (Sheldrick, 2015*b* ▸), *DIAMOND* (Brandenburg & Putz, 2012 ▸) and *SHELXTL* (Sheldrick, 2008 ▸).

## supplementary crystallographic information

### Crystal data

**Table:**

C_16_H_20_N_2_O_2_	*Z* = 4
*M**_r_* = 272.34	*F*(000) = 584
Triclinic, *P*1	*D*_x_ = 1.249 Mg m^−^^3^
*a* = 9.1132 (6) Å	Mo *K*α radiation, λ = 0.71073 Å
*b* = 12.6676 (9) Å	Cell parameters from 9901 reflections
*c* = 12.8164 (9) Å	θ = 2.2–29.6°
α = 91.344 (1)°	µ = 0.08 mm^−^^1^
β = 99.537 (1)°	*T* = 100 K
γ = 96.340 (1)°	Block, colourless
*V* = 1448.87 (17) Å^3^	0.34 × 0.28 × 0.25 mm

### Data collection

**Table:**

Bruker SMART APEX CCD diffractometer	51542 independent reflections
Radiation source: fine-focus sealed tube	40020 reflections with *I* > 2σ(*I*)
Graphite monochromator	*R*_int_ = 0.029
Detector resolution: 8.3333 pixels mm^-1^	θ_max_ = 29.7°, θ_min_ = 1.6°
φ and ω scans	*h* = −12→12
Absorption correction: multi-scan (*TWINABS*; Sheldrick, 2009)	*k* = −17→17
*T*_min_ = 0.97, *T*_max_ = 0.98	*l* = −17→17
51542 measured reflections	

### Refinement

**Table:**

Refinement on *F*^2^	Primary atom site location: structure-invariant direct methods
Least-squares matrix: full	Secondary atom site location: difference Fourier map
*R*[*F*^2^ > 2σ(*F*^2^)] = 0.045	Hydrogen site location: mixed
*wR*(*F*^2^) = 0.133	H atoms treated by a mixture of independent and constrained refinement
*S* = 1.08	*w* = 1/[σ^2^(*F*_o_^2^) + (0.0752*P*)^2^ + 0.0382*P*] where *P* = (*F*_o_^2^ + 2*F*_c_^2^)/3
51542 reflections	(Δ/σ)_max_ = 0.001
511 parameters	Δρ_max_ = 0.41 e Å^−^^3^
0 restraints	Δρ_min_ = −0.32 e Å^−^^3^

### Special details

**Table:**

Experimental. The diffraction data were obtained from 3 sets of 400 frames, each of width 0.5° in ω, colllected at φ = 0.00, 90.00 and 180.00° and 2 sets of 800 frames, each of width 0.45° in φ, collected at ω = –30.00 and 210.00°. The scan time was 20 sec/frame. Analysis of 641 reflections having I/σ(I) > 13 and chosen from the full data set with *CELL_NOW* (Sheldrick, 2008) showed the crystal to belong to the triclinic system and to be twinned by a 180° rotation about the reciprocal axis [111]. The raw data were processed using the multi-component version of *SAINT* under control of the two-component orientation file generated by *CELL_NOW*.
Geometry. All esds (except the esd in the dihedral angle between two l.s. planes) are estimated using the full covariance matrix. The cell esds are taken into account individually in the estimation of esds in distances, angles and torsion angles; correlations between esds in cell parameters are only used when they are defined by crystal symmetry. An approximate (isotropic) treatment of cell esds is used for estimating esds involving l.s. planes.
Refinement. Refinement of F^2^ against ALL reflections. The weighted R-factor wR and goodness of fit S are based on F^2^, conventional R-factors R are based on F, with F set to zero for negative F^2^. The threshold expression of F^2^ > 2sigma(F^2^) is used only for calculating R-factors(gt) etc. and is not relevant to the choice of reflections for refinement. R-factors based on F^2^ are statistically about twice as large as those based on F, and R- factors based on ALL data will be even larger. Refined as a 2-component twin. Individual refinement of the H-atoms attached to C28 did not give a satisfactory geometry so these were included as riding contributions in idealized positions.

### Fractional atomic coordinates and isotropic or equivalent isotropic displacement parameters (Å^2^)

**Table:**

	*x*	*y*	*z*	*U*_iso_*/*U*_eq_	
O1	0.72177 (11)	0.04051 (8)	0.54430 (7)	0.0238 (2)	
O2	0.56968 (10)	0.43831 (7)	0.35257 (7)	0.0215 (2)	
N1	0.72935 (12)	0.28280 (9)	0.33604 (8)	0.0158 (2)	
H1	0.7127 (18)	0.3534 (13)	0.3296 (12)	0.032 (4)*	
N2	0.89478 (12)	0.13227 (8)	0.46108 (8)	0.0165 (2)	
C1	0.93805 (14)	0.17354 (10)	0.36609 (9)	0.0157 (3)	
C2	1.06770 (15)	0.14365 (11)	0.33411 (10)	0.0185 (3)	
H2	1.1192 (17)	0.0914 (12)	0.3745 (11)	0.023 (4)*	
C3	1.11875 (15)	0.18668 (11)	0.24671 (11)	0.0204 (3)	
H3	1.2087 (17)	0.1646 (11)	0.2263 (11)	0.023 (4)*	
C4	1.04086 (15)	0.26135 (11)	0.18968 (11)	0.0208 (3)	
H4	1.0748 (17)	0.2907 (12)	0.1285 (12)	0.023 (4)*	
C5	0.91218 (15)	0.29138 (11)	0.21946 (10)	0.0185 (3)	
H5	0.8544 (18)	0.3453 (12)	0.1809 (12)	0.026 (4)*	
C6	0.85894 (14)	0.24746 (10)	0.30730 (9)	0.0155 (3)	
C7	0.61586 (14)	0.22068 (10)	0.36742 (9)	0.0151 (3)	
C8	0.63211 (15)	0.10416 (10)	0.37404 (10)	0.0178 (3)	
H8A	0.6596 (17)	0.0781 (12)	0.3060 (11)	0.023 (4)*	
H8B	0.5373 (17)	0.0641 (12)	0.3867 (11)	0.023 (4)*	
C9	0.75241 (15)	0.08784 (10)	0.46686 (10)	0.0169 (3)	
C10	0.49225 (14)	0.26264 (10)	0.39233 (9)	0.0162 (3)	
H10	0.4120 (17)	0.2170 (12)	0.4145 (11)	0.023 (4)*	
C11	0.47391 (14)	0.37296 (10)	0.38320 (9)	0.0171 (3)	
C12	0.33482 (16)	0.41213 (12)	0.41142 (12)	0.0235 (3)	
H12A	0.243 (2)	0.3606 (15)	0.3887 (14)	0.046 (5)*	
H12B	0.321 (2)	0.4819 (14)	0.3787 (13)	0.038 (5)*	
H12C	0.348 (2)	0.4220 (14)	0.4879 (14)	0.045 (5)*	
C13	1.01107 (16)	0.13553 (11)	0.55643 (10)	0.0214 (3)	
H13A	1.086 (2)	0.0890 (13)	0.5451 (13)	0.038 (5)*	
H13B	0.9588 (16)	0.1036 (11)	0.6123 (11)	0.019 (4)*	
C14	1.08024 (16)	0.24803 (11)	0.59066 (11)	0.0214 (3)	
H14A	1.1376 (17)	0.2780 (12)	0.5385 (11)	0.023 (4)*	
H14B	1.1531 (19)	0.2442 (12)	0.6570 (13)	0.031 (4)*	
C15	0.96927 (17)	0.32465 (11)	0.60967 (12)	0.0246 (3)	
H15A	0.899 (2)	0.3314 (13)	0.5431 (13)	0.038 (5)*	
H15B	0.9065 (19)	0.2921 (13)	0.6622 (13)	0.036 (4)*	
C16	1.0457 (2)	0.43481 (13)	0.64748 (14)	0.0331 (4)	
H16A	1.109 (2)	0.4651 (14)	0.5958 (14)	0.045 (5)*	
H16B	1.113 (2)	0.4355 (15)	0.7181 (15)	0.051 (5)*	
H16C	0.973 (2)	0.4854 (15)	0.6559 (13)	0.044 (5)*	
O3	0.58897 (11)	0.87290 (7)	−0.13509 (7)	0.0202 (2)	
O4	0.79438 (11)	0.48603 (7)	0.05767 (7)	0.0237 (2)	
N3	0.63297 (12)	0.64210 (9)	0.08256 (8)	0.0159 (2)	
H3A	0.690 (2)	0.5925 (14)	0.1094 (13)	0.035 (5)*	
N4	0.58830 (12)	0.86428 (8)	0.04194 (8)	0.0145 (2)	
C17	0.54349 (14)	0.81037 (10)	0.13050 (9)	0.0148 (3)	
C18	0.47824 (15)	0.86709 (11)	0.20216 (10)	0.0182 (3)	
H18	0.4569 (16)	0.9382 (12)	0.1862 (11)	0.020 (4)*	
C19	0.44752 (16)	0.82329 (11)	0.29503 (10)	0.0218 (3)	
H19	0.4001 (18)	0.8658 (12)	0.3443 (12)	0.028 (4)*	
C20	0.48108 (16)	0.72094 (11)	0.31750 (10)	0.0216 (3)	
H20	0.4601 (18)	0.6882 (12)	0.3839 (12)	0.028 (4)*	
C21	0.54102 (15)	0.66222 (11)	0.24625 (10)	0.0189 (3)	
H21	0.5639 (17)	0.5889 (12)	0.2591 (11)	0.025 (4)*	
C22	0.57102 (14)	0.70569 (10)	0.15174 (9)	0.0152 (3)	
C23	0.59693 (14)	0.63618 (10)	−0.02420 (9)	0.0157 (3)	
C24	0.48707 (15)	0.70842 (10)	−0.07315 (10)	0.0169 (3)	
H24A	0.4620 (17)	0.6921 (11)	−0.1483 (12)	0.022 (4)*	
H24B	0.3985 (17)	0.7000 (11)	−0.0395 (11)	0.018 (4)*	
C25	0.55790 (13)	0.82264 (10)	−0.05945 (10)	0.0152 (3)	
C26	0.65922 (15)	0.56812 (10)	−0.08471 (10)	0.0180 (3)	
H26	0.6285 (17)	0.5668 (11)	−0.1622 (11)	0.020 (4)*	
C27	0.76044 (14)	0.49577 (10)	−0.03993 (11)	0.0198 (3)	
C28	0.82882 (17)	0.42914 (12)	−0.11377 (12)	0.0292 (3)	
H28A	0.773193	0.430696	−0.185929	0.044*	
H28B	0.824151	0.355681	−0.091077	0.044*	
H28C	0.933585	0.457712	−0.112321	0.044*	
C29	0.68172 (15)	0.96788 (10)	0.06506 (11)	0.0178 (3)	
H29A	0.6920 (15)	0.9984 (11)	−0.0031 (11)	0.013 (3)*	
H29B	0.6320 (16)	1.0178 (11)	0.1058 (11)	0.018 (4)*	
C30	0.83516 (15)	0.95516 (11)	0.12782 (11)	0.0189 (3)	
H30A	0.8940 (17)	1.0277 (12)	0.1407 (11)	0.025 (4)*	
H30B	0.8225 (17)	0.9296 (11)	0.2017 (11)	0.021 (4)*	
C31	0.92392 (15)	0.88366 (11)	0.07175 (12)	0.0223 (3)	
H31A	0.9269 (17)	0.9081 (12)	−0.0022 (12)	0.026 (4)*	
H31B	0.8703 (18)	0.8104 (13)	0.0616 (12)	0.028 (4)*	
C32	1.08216 (17)	0.87977 (14)	0.13147 (14)	0.0300 (3)	
H32A	1.080 (2)	0.8497 (14)	0.2012 (15)	0.045 (5)*	
H32B	1.1370 (19)	0.9530 (14)	0.1406 (12)	0.034 (4)*	
H32C	1.1392 (19)	0.8371 (13)	0.0902 (13)	0.038 (5)*	

### Atomic displacement parameters (Å^2^)

**Table:**

	*U*^11^	*U*^22^	*U*^33^	*U*^12^	*U*^13^	*U*^23^
O1	0.0278 (5)	0.0238 (5)	0.0237 (5)	0.0096 (4)	0.0103 (4)	0.0092 (4)
O2	0.0236 (5)	0.0172 (5)	0.0250 (5)	0.0042 (4)	0.0056 (4)	0.0058 (4)
N1	0.0169 (5)	0.0140 (5)	0.0174 (5)	0.0042 (4)	0.0037 (4)	0.0030 (4)
N2	0.0200 (6)	0.0153 (5)	0.0140 (5)	0.0031 (4)	0.0011 (4)	0.0020 (4)
C1	0.0178 (6)	0.0144 (6)	0.0138 (6)	0.0003 (5)	0.0010 (5)	−0.0004 (5)
C2	0.0174 (6)	0.0151 (6)	0.0219 (7)	0.0024 (5)	−0.0002 (5)	−0.0013 (5)
C3	0.0162 (6)	0.0208 (7)	0.0239 (7)	−0.0001 (5)	0.0044 (5)	−0.0056 (5)
C4	0.0212 (7)	0.0220 (7)	0.0184 (6)	−0.0035 (5)	0.0050 (5)	−0.0009 (5)
C5	0.0199 (6)	0.0176 (7)	0.0168 (6)	0.0005 (5)	0.0006 (5)	0.0014 (5)
C6	0.0152 (6)	0.0149 (6)	0.0159 (6)	0.0013 (5)	0.0019 (5)	−0.0015 (5)
C7	0.0176 (6)	0.0156 (6)	0.0112 (5)	0.0020 (5)	−0.0006 (5)	0.0010 (5)
C8	0.0192 (6)	0.0150 (6)	0.0192 (6)	0.0015 (5)	0.0036 (5)	0.0008 (5)
C9	0.0220 (7)	0.0123 (6)	0.0181 (6)	0.0065 (5)	0.0056 (5)	0.0006 (5)
C10	0.0164 (6)	0.0167 (6)	0.0150 (6)	0.0015 (5)	0.0015 (5)	0.0014 (5)
C11	0.0192 (6)	0.0196 (7)	0.0123 (6)	0.0040 (5)	0.0007 (5)	0.0009 (5)
C12	0.0220 (7)	0.0217 (7)	0.0282 (8)	0.0067 (6)	0.0061 (6)	−0.0003 (6)
C13	0.0241 (7)	0.0225 (7)	0.0169 (6)	0.0073 (6)	−0.0019 (6)	0.0028 (5)
C14	0.0189 (7)	0.0242 (7)	0.0198 (7)	0.0021 (5)	−0.0001 (6)	−0.0001 (5)
C15	0.0246 (7)	0.0225 (7)	0.0254 (7)	0.0026 (6)	0.0013 (6)	−0.0023 (6)
C16	0.0341 (9)	0.0256 (8)	0.0373 (9)	0.0006 (7)	0.0024 (8)	−0.0083 (7)
O3	0.0272 (5)	0.0192 (5)	0.0164 (4)	0.0063 (4)	0.0071 (4)	0.0050 (4)
O4	0.0235 (5)	0.0206 (5)	0.0277 (5)	0.0055 (4)	0.0039 (4)	0.0050 (4)
N3	0.0187 (5)	0.0137 (5)	0.0155 (5)	0.0040 (4)	0.0016 (4)	0.0022 (4)
N4	0.0161 (5)	0.0137 (5)	0.0139 (5)	0.0013 (4)	0.0028 (4)	0.0030 (4)
C17	0.0141 (6)	0.0161 (6)	0.0134 (6)	−0.0004 (5)	0.0014 (5)	0.0028 (5)
C18	0.0182 (6)	0.0169 (7)	0.0192 (6)	0.0017 (5)	0.0028 (5)	0.0006 (5)
C19	0.0222 (7)	0.0262 (8)	0.0172 (6)	0.0012 (6)	0.0059 (6)	−0.0024 (5)
C20	0.0236 (7)	0.0259 (7)	0.0141 (6)	−0.0027 (6)	0.0032 (5)	0.0031 (5)
C21	0.0204 (7)	0.0178 (7)	0.0165 (6)	−0.0013 (5)	−0.0005 (5)	0.0032 (5)
C22	0.0134 (6)	0.0164 (6)	0.0145 (6)	0.0001 (5)	0.0003 (5)	0.0002 (5)
C23	0.0157 (6)	0.0131 (6)	0.0171 (6)	−0.0018 (5)	0.0014 (5)	0.0025 (5)
C24	0.0171 (6)	0.0178 (7)	0.0148 (6)	0.0017 (5)	−0.0002 (5)	0.0008 (5)
C25	0.0136 (6)	0.0162 (6)	0.0168 (6)	0.0060 (5)	0.0023 (5)	0.0027 (5)
C26	0.0196 (6)	0.0165 (6)	0.0174 (6)	−0.0010 (5)	0.0034 (5)	0.0003 (5)
C27	0.0159 (6)	0.0154 (6)	0.0271 (7)	−0.0025 (5)	0.0043 (5)	−0.0005 (5)
C28	0.0256 (7)	0.0271 (8)	0.0362 (8)	0.0070 (6)	0.0071 (6)	−0.0053 (6)
C29	0.0202 (6)	0.0132 (6)	0.0206 (6)	0.0013 (5)	0.0056 (5)	0.0016 (5)
C30	0.0185 (6)	0.0180 (7)	0.0191 (6)	−0.0017 (5)	0.0024 (5)	0.0002 (5)
C31	0.0185 (7)	0.0195 (7)	0.0289 (8)	0.0015 (5)	0.0047 (6)	0.0005 (6)
C32	0.0204 (7)	0.0333 (9)	0.0368 (9)	0.0054 (7)	0.0036 (7)	0.0089 (7)

### Geometric parameters (Å, º)

**Table:**

O1—C9	1.2269 (14)	O3—C25	1.2271 (14)
O2—C11	1.2532 (15)	O4—C27	1.2503 (16)
N1—C7	1.3504 (16)	N3—C23	1.3515 (16)
N1—C6	1.4086 (15)	N3—C22	1.4095 (15)
N1—H1	0.927 (17)	N3—H3A	0.898 (17)
N2—C9	1.3704 (16)	N4—C25	1.3637 (15)
N2—C1	1.4333 (15)	N4—C17	1.4316 (14)
N2—C13	1.4755 (17)	N4—C29	1.4786 (16)
C1—C2	1.3998 (17)	C17—C18	1.3993 (17)
C1—C6	1.4023 (18)	C17—C22	1.4011 (17)
C2—C3	1.3833 (18)	C18—C19	1.3820 (18)
C2—H2	0.964 (15)	C18—H18	0.961 (14)
C3—C4	1.3935 (19)	C19—C20	1.3905 (19)
C3—H3	0.968 (15)	C19—H19	1.001 (15)
C4—C5	1.3804 (18)	C20—C21	1.3816 (18)
C4—H4	0.958 (14)	C20—H20	0.993 (14)
C5—C6	1.4012 (17)	C21—C22	1.3974 (16)
C5—H5	0.998 (16)	C21—H21	0.986 (15)
C7—C10	1.3772 (17)	C23—C26	1.3746 (17)
C7—C8	1.5025 (17)	C23—C24	1.5014 (17)
C8—C9	1.5131 (19)	C24—C25	1.5118 (18)
C8—H8A	1.005 (14)	C24—H24A	0.964 (15)
C8—H8B	0.992 (15)	C24—H24B	0.974 (14)
C10—C11	1.4309 (17)	C26—C27	1.4339 (18)
C10—H10	0.966 (15)	C26—H26	0.986 (14)
C11—C12	1.5054 (18)	C27—C28	1.5068 (18)
C12—H12A	0.999 (19)	C28—H28A	0.9800
C12—H12B	0.999 (17)	C28—H28B	0.9800
C12—H12C	0.971 (18)	C28—H28C	0.9800
C13—C14	1.5170 (19)	C29—C30	1.5203 (19)
C13—H13A	0.979 (17)	C29—H29A	0.977 (13)
C13—H13B	0.993 (13)	C29—H29B	1.002 (14)
C14—C15	1.5204 (19)	C30—C31	1.5215 (18)
C14—H14A	0.974 (14)	C30—H30A	1.007 (16)
C14—H14B	0.994 (17)	C30—H30B	1.028 (14)
C15—C16	1.520 (2)	C31—C32	1.523 (2)
C15—H15A	0.990 (18)	C31—H31A	1.008 (14)
C15—H15B	1.020 (16)	C31—H31B	0.995 (16)
C16—H16A	1.005 (18)	C32—H32A	0.982 (17)
C16—H16B	1.00 (2)	C32—H32B	0.998 (18)
C16—H16C	0.986 (19)	C32—H32C	0.990 (17)
			
O1···H19^i^	2.328 (16)	C11···H26^iv^	2.976 (14)
O1···H13A^ii^	2.878 (18)	C13···H2	2.746 (15)
O1···H2^ii^	2.468 (15)	C17···H24B	2.635 (14)
O1···H13B	2.242 (15)	C17···H30B	2.810 (15)
O1···H8B^iii^	2.858 (15)	C18···H29B	2.688 (14)
O2···H21	2.277 (15)	C21···H14B^ix^	2.961 (17)
O2···H12C^i^	2.627 (18)	C21···H28A^iv^	2.94
O2···H1	1.834 (17)	C22···H24B	2.679 (14)
O2···H26^iv^	2.780 (14)	C25···H29B^v^	2.815 (14)
O3···H18^v^	2.556 (15)	C26···H4^vi^	2.988 (16)
O3···H3^vi^	2.424 (15)	C27···H3A	2.459 (17)
O3···H29B^v^	2.637 (15)	C29···H18	2.768 (15)
O3···H29A	2.300 (14)	H1···H5	2.48 (2)
O4···H3A	1.901 (18)	H2···H13A	2.26 (2)
O4···H24B^iv^	2.761 (14)	H2···H13B^ii^	2.51 (2)
O4···H5	2.456 (15)	H3A···H21	2.39 (2)
O4···H28C^vi^	2.48	H8B···H10	2.40 (2)
N1···H24A^iv^	2.775 (15)	H10···H12A	2.50 (2)
N1···H15A	2.858 (17)	H12A···H14A^x^	2.49 (2)
N2···H13A^ii^	2.828 (16)	H13B···H32A^ix^	2.54 (2)
N2···H15A	2.704 (16)	H13B···H15B	2.57 (2)
N3···H31B	2.915 (17)	H14A···H16A	2.52 (2)
N4···H31B	2.705 (17)	H15B···H32A^ix^	2.54 (2)
C1···H8A	2.669 (16)	H18···H29B	2.21 (2)
C1···H14A	2.818 (15)	H24A···H26	2.34 (2)
C2···H13A	2.790 (16)	H26···H28A	2.33
C2···H30A^vii^	2.970 (14)	H28B···H32C^vi^	2.50
C3···H12A^viii^	2.833 (19)	H29A···H31A	2.54 (2)
C3···H30A^vii^	2.852 (15)	H29A···H32B^xi^	2.58 (2)
C6···H8A	2.652 (15)	H30A···H32B	2.50 (2)
C9···H13A^ii^	2.828 (17)	H31A···H31A^xi^	2.55 (2)
C10···H20^i^	2.871 (15)	H31A···H32B^xi^	2.57 (2)
C11···H1	2.424 (17)	H32C···H28B^vi^	2.50
			
C7—N1—C6	125.71 (11)	C23—N3—C22	125.86 (11)
C7—N1—H1	113.8 (10)	C23—N3—H3A	114.1 (10)
C6—N1—H1	120.3 (10)	C22—N3—H3A	119.2 (10)
C9—N2—C1	123.36 (10)	C25—N4—C17	123.89 (10)
C9—N2—C13	118.67 (10)	C25—N4—C29	119.17 (10)
C1—N2—C13	117.95 (10)	C17—N4—C29	116.80 (10)
C2—C1—C6	118.91 (11)	C18—C17—C22	118.86 (11)
C2—C1—N2	119.06 (11)	C18—C17—N4	118.36 (11)
C6—C1—N2	121.94 (10)	C22—C17—N4	122.65 (10)
C3—C2—C1	120.99 (12)	C19—C18—C17	121.09 (12)
C3—C2—H2	121.3 (8)	C19—C18—H18	120.4 (8)
C1—C2—H2	117.7 (8)	C17—C18—H18	118.5 (8)
C2—C3—C4	119.77 (12)	C18—C19—C20	119.57 (12)
C2—C3—H3	119.3 (8)	C18—C19—H19	119.0 (8)
C4—C3—H3	121.0 (8)	C20—C19—H19	121.4 (8)
C5—C4—C3	120.16 (12)	C21—C20—C19	120.30 (12)
C5—C4—H4	119.6 (9)	C21—C20—H20	118.9 (9)
C3—C4—H4	120.2 (9)	C19—C20—H20	120.7 (9)
C4—C5—C6	120.43 (13)	C20—C21—C22	120.39 (13)
C4—C5—H5	122.2 (8)	C20—C21—H21	122.3 (8)
C6—C5—H5	117.4 (8)	C22—C21—H21	117.3 (8)
C5—C6—C1	119.73 (11)	C21—C22—C17	119.69 (11)
C5—C6—N1	118.21 (11)	C21—C22—N3	117.74 (11)
C1—C6—N1	122.02 (11)	C17—C22—N3	122.54 (11)
N1—C7—C10	121.39 (11)	N3—C23—C26	121.48 (12)
N1—C7—C8	116.55 (11)	N3—C23—C24	116.67 (11)
C10—C7—C8	122.07 (11)	C26—C23—C24	121.85 (11)
C7—C8—C9	109.20 (11)	C23—C24—C25	109.99 (10)
C7—C8—H8A	108.7 (8)	C23—C24—H24A	109.3 (9)
C9—C8—H8A	110.6 (9)	C25—C24—H24A	106.8 (9)
C7—C8—H8B	110.3 (9)	C23—C24—H24B	109.5 (8)
C9—C8—H8B	107.5 (8)	C25—C24—H24B	109.8 (8)
H8A—C8—H8B	110.5 (12)	H24A—C24—H24B	111.4 (13)
O1—C9—N2	122.40 (12)	O3—C25—N4	122.76 (11)
O1—C9—C8	121.18 (11)	O3—C25—C24	121.48 (11)
N2—C9—C8	116.38 (11)	N4—C25—C24	115.73 (10)
C7—C10—C11	122.38 (12)	C23—C26—C27	123.00 (12)
C7—C10—H10	120.3 (9)	C23—C26—H26	118.0 (8)
C11—C10—H10	117.3 (9)	C27—C26—H26	118.9 (8)
O2—C11—C10	122.32 (11)	O4—C27—C26	122.69 (11)
O2—C11—C12	118.72 (12)	O4—C27—C28	118.82 (12)
C10—C11—C12	118.95 (12)	C26—C27—C28	118.50 (12)
C11—C12—H12A	112.7 (10)	C27—C28—H28A	109.5
C11—C12—H12B	109.2 (10)	C27—C28—H28B	109.5
H12A—C12—H12B	110.0 (15)	H28A—C28—H28B	109.5
C11—C12—H12C	108.3 (11)	C27—C28—H28C	109.5
H12A—C12—H12C	107.4 (14)	H28A—C28—H28C	109.5
H12B—C12—H12C	109.1 (15)	H28B—C28—H28C	109.5
N2—C13—C14	112.25 (11)	N4—C29—C30	111.51 (10)
N2—C13—H13A	110.0 (10)	N4—C29—H29A	106.9 (8)
C14—C13—H13A	112.2 (10)	C30—C29—H29A	110.2 (8)
N2—C13—H13B	105.2 (8)	N4—C29—H29B	111.1 (8)
C14—C13—H13B	110.1 (8)	C30—C29—H29B	108.8 (8)
H13A—C13—H13B	106.6 (12)	H29A—C29—H29B	108.2 (11)
C13—C14—C15	114.98 (12)	C29—C30—C31	113.61 (11)
C13—C14—H14A	110.6 (9)	C29—C30—H30A	108.1 (9)
C15—C14—H14A	107.9 (9)	C31—C30—H30A	108.5 (8)
C13—C14—H14B	107.6 (9)	C29—C30—H30B	108.9 (8)
C15—C14—H14B	109.0 (9)	C31—C30—H30B	112.1 (8)
H14A—C14—H14B	106.4 (13)	H30A—C30—H30B	105.2 (11)
C16—C15—C14	112.47 (13)	C30—C31—C32	112.70 (13)
C16—C15—H15A	108.2 (10)	C30—C31—H31A	109.6 (8)
C14—C15—H15A	109.5 (10)	C32—C31—H31A	110.4 (9)
C16—C15—H15B	111.8 (9)	C30—C31—H31B	109.8 (9)
C14—C15—H15B	108.1 (9)	C32—C31—H31B	109.4 (9)
H15A—C15—H15B	106.6 (14)	H31A—C31—H31B	104.6 (12)
C15—C16—H16A	111.0 (11)	C31—C32—H32A	110.7 (11)
C15—C16—H16B	113.0 (11)	C31—C32—H32B	109.7 (10)
H16A—C16—H16B	107.2 (16)	H32A—C32—H32B	109.6 (14)
C15—C16—H16C	112.3 (11)	C31—C32—H32C	110.6 (10)
H16A—C16—H16C	107.2 (14)	H32A—C32—H32C	109.8 (14)
H16B—C16—H16C	105.7 (14)	H32B—C32—H32C	106.4 (13)
			
C9—N2—C1—C2	−131.39 (13)	C25—N4—C17—C18	−133.96 (13)
C13—N2—C1—C2	46.95 (15)	C29—N4—C17—C18	50.34 (15)
C9—N2—C1—C6	52.13 (16)	C25—N4—C17—C22	50.23 (17)
C13—N2—C1—C6	−129.53 (13)	C29—N4—C17—C22	−125.47 (13)
C6—C1—C2—C3	0.67 (19)	C22—C17—C18—C19	3.05 (19)
N2—C1—C2—C3	−175.92 (11)	N4—C17—C18—C19	−172.93 (12)
C1—C2—C3—C4	0.34 (19)	C17—C18—C19—C20	−0.4 (2)
C2—C3—C4—C5	−0.78 (19)	C18—C19—C20—C21	−1.7 (2)
C3—C4—C5—C6	0.20 (19)	C19—C20—C21—C22	1.1 (2)
C4—C5—C6—C1	0.82 (19)	C20—C21—C22—C17	1.57 (19)
C4—C5—C6—N1	178.38 (12)	C20—C21—C22—N3	179.63 (12)
C2—C1—C6—C5	−1.24 (18)	C18—C17—C22—C21	−3.61 (18)
N2—C1—C6—C5	175.25 (11)	N4—C17—C22—C21	172.18 (11)
C2—C1—C6—N1	−178.70 (11)	C18—C17—C22—N3	178.43 (12)
N2—C1—C6—N1	−2.21 (18)	N4—C17—C22—N3	−5.78 (19)
C7—N1—C6—C5	138.62 (13)	C23—N3—C22—C21	140.48 (13)
C7—N1—C6—C1	−43.88 (18)	C23—N3—C22—C17	−41.51 (19)
C6—N1—C7—C10	−178.93 (12)	C22—N3—C23—C26	−177.17 (12)
C6—N1—C7—C8	1.32 (18)	C22—N3—C23—C24	3.50 (18)
N1—C7—C8—C9	70.83 (13)	N3—C23—C24—C25	69.03 (14)
C10—C7—C8—C9	−108.91 (13)	C26—C23—C24—C25	−110.29 (13)
C1—N2—C9—O1	170.18 (11)	C17—N4—C25—O3	175.20 (11)
C13—N2—C9—O1	−8.15 (17)	C29—N4—C25—O3	−9.19 (17)
C1—N2—C9—C8	−12.36 (16)	C17—N4—C25—C24	−6.60 (16)
C13—N2—C9—C8	169.31 (10)	C29—N4—C25—C24	169.00 (10)
C7—C8—C9—O1	113.48 (13)	C23—C24—C25—O3	110.58 (13)
C7—C8—C9—N2	−64.01 (13)	C23—C24—C25—N4	−67.64 (13)
N1—C7—C10—C11	2.17 (19)	N3—C23—C26—C27	2.88 (19)
C8—C7—C10—C11	−178.09 (11)	C24—C23—C26—C27	−177.82 (11)
C7—C10—C11—O2	−0.45 (19)	C23—C26—C27—O4	3.2 (2)
C7—C10—C11—C12	179.61 (12)	C23—C26—C27—C28	−176.60 (12)
C9—N2—C13—C14	−121.28 (12)	C25—N4—C29—C30	−109.33 (12)
C1—N2—C13—C14	60.30 (14)	C17—N4—C29—C30	66.58 (13)
N2—C13—C14—C15	55.44 (16)	N4—C29—C30—C31	59.09 (14)
C13—C14—C15—C16	177.96 (13)	C29—C30—C31—C32	174.97 (12)

Symmetry codes: (i) −*x*+1, −*y*+1, −*z*+1; (ii) −*x*+2, −*y*, −*z*+1; (iii) −*x*+1, −*y*, −*z*+1; (iv) −*x*+1, −*y*+1, −*z*; (v) −*x*+1, −*y*+2, −*z*; (vi) −*x*+2, −*y*+1, −*z*; (vii) *x*, *y*−1, *z*; (viii) *x*+1, *y*, *z*; (ix) −*x*+2, −*y*+1, −*z*+1; (x) *x*−1, *y*, *z*; (xi) −*x*+2, −*y*+2, −*z*.

### Hydrogen-bond geometry (Å, º)

Cg1 is the centroid of benzene ring *A* (C1–C6).

**Table:**

*D*—H···*A*	*D*—H	H···*A*	*D*···*A*	*D*—H···*A*
N1—H1···O2	0.927 (17)	1.834 (17)	2.5998 (14)	138.2 (13)
N3—H3*A*···O4	0.898 (17)	1.901 (17)	2.6349 (14)	137.6 (14)
C2—H2···O1^ii^	0.964 (15)	2.469 (16)	3.4235 (17)	170.6 (11)
C3—H3···O3^vi^	0.968 (15)	2.420 (17)	3.3714 (16)	166.0 (11)
C5—H5···O4	0.998 (16)	2.456 (15)	3.4086 (17)	159.3 (11)
C18—H18···O3^v^	0.961 (14)	2.556 (15)	3.5165 (16)	176.4 (11)
C19—H19···O1^i^	1.001 (15)	2.330 (15)	3.3273 (15)	177.0 (12)
C21—H21···O2	0.986 (15)	2.277 (15)	3.1933 (16)	154.1 (11)
C28—H28*C*···O4^vi^	0.98	2.48	3.4342 (18)	164
C12—H12*A*···*Cg*1^x^	0.999 (19)	2.921 (19)	3.9047 (16)	167.8 (13)
C30—H30*A*···*Cg*1^xii^	1.007 (16)	2.903 (15)	3.8016 (15)	149.0 (11)

Symmetry codes: (i) −*x*+1, −*y*+1, −*z*+1; (ii) −*x*+2, −*y*, −*z*+1; (v) −*x*+1, −*y*+2, −*z*; (vi) −*x*+2, −*y*+1, −*z*; (x) *x*−1, *y*, *z*; (xii) *x*, *y*+1, *z*.
